# Purification of Flavonoids from an Aqueous Cocoa (*Theobroma cocoa* L.) Extract Using Macroporous Adsorption Resins

**DOI:** 10.3390/molecules30112336

**Published:** 2025-05-27

**Authors:** Nicole Beeler, Tilo Hühn, Sascha Rohn, Renato Colombi

**Affiliations:** 1 Food Process Development Research Group, Institute of Food and Beverage Innovation, School of Life Sciences and Facility Management, Zurich University of Applied Sciences, 8820 Wädenswil, Switzerland; tilo.huehn@zhaw.ch; 2 Department of Food Chemistry and Analysis, Institute of Food Technology and Food Chemistry, Technische Universität Berlin, 13355 Berlin, Germany; rohn@tu-berlin.de; 3 Oro de Cacao AG, Chocolate Manufacturer, 8807 Freienbach, Switzerland; renato.colombi@orodecacao.com

**Keywords:** cocoa extract, cocoa flavonoids, purification, adsorption, desorption

## Abstract

Cocoa is a rich source of health-promoting polyphenols such as flavanols. These compounds can be separated from other matrix constituents using various adsorbents or resins. Seven different macroporous resins (Amberlite^®^ XAD-2, XAD-4, XAD-7, XAD-7HP, XAD-16, Sepabeads^TM^ SP207, and Diaion^®^ HP2-MG) were evaluated for their adsorption and desorption properties for the enrichment of flavonoids from an aqueous cocoa (*Theobroma cacao* L.) extract. The influence of adsorption and desorption temperatures and the concentration of the desorption solvent (a hydroalcoholic solution) were investigated by static adsorption and desorption methods. The results of the resin comparison showed that the adsorbent XAD-7HP had the best adsorption characteristics, with an adsorption capacity of 39.8 mg ECE/g. XAD-7HP was found to be the most suitable adsorbent, and 70% ethanol was the best desorbing solvent, based on static experiments. In addition, the optimal conditions for the adsorption of flavonoids were obtained at a temperature of 30 °C, where equilibrium was reached after 80 min. The static adsorption process was well-described by a pseudo-second-order kinetics model, while the adsorption isotherm data were fitted well by the Freundlich isotherm model. Further dynamic adsorption and desorption characteristics were evaluated on a packed glass column, and it was shown that XAD-7HP could enrich the flavanol content by 5.03-fold, with a dry matter content of 456.05 mg/mL (as estimated by the degree of DP1–DP7 procyanidin polymers using ultra-pressure liquid chromatography).

## 1. Introduction

Bioactive compounds in various plant foods (and by-products) have attracted considerable interest in recent years due to their health-promoting properties, which have been demonstrated in numerous studies [1,2]. Among these, the seeds of *Theobroma cacao* L. are often highlighted, as these are particularly rich in polyphenols. These polyphenols are mainly monomeric flavan-3-ols and their oligomers, called procyanidins. The monomeric (+)-catechin and (−)-epicatechin constitute about one-third of the total flavan-3-ols, while procyanidins make up the rest [3].

In this context, chocolate and cocoa-containing products are consequently a source of those bioactive polyphenols, bearing potential health benefits as well [3]. Significant efforts have been made to valorize by-products and wastes from the food industry by recovering bioactive substances for re-application in foods, cosmetics, or pharmaceuticals [1,2]. To meet the steadily increasing demand, the development of simple and efficient methods for the separation and purification of cocoa flavonoids is essential. As the main limitation, impurities in the extracts often compromise their biological efficacy, further highlighting the need for efficient purification methods to target and isolate bioactive compounds [4]. Traditional methods for flavonoid purification, including solid–liquid extraction, liquid–liquid extraction, and column chromatography, often face typical challenges, such as high costs, long processing times, and scalability issues [5,6,7,8]. Adsorption using macroporous adsorption resins has emerged as an environmentally friendly and effective alternative for the separation and purification of bioactive substances [9]. Macroporous resins enable reversible adsorption, allowing for the efficient recovery and elution of target compounds, often using ethanol–water solutions as safe and effective solvents [10,11]. In recent years, macroporous resins have been further improved to recover and concentrate bioactive compounds from food industry by-products, such as citrus peels, molasses, and fruit pomaces [12,13,14].

The most prominent macroporous resins consist of highly crosslinked polymer beads with large surface areas and defined porosities, offering high adsorption capacity, easy regenerability, and chemical stability. Their adsorption mechanisms are based on specific interactions, such as hydrogen bonding between the hydroxyl groups of phenolic compounds and the carboxyl groups of the resin. This makes macroporous resins particularly effective for purifying flavonoids, which are characterized by aromatic rings and hydroxyl groups [6,7,8,15,16,17,18]. Compared to other separation techniques, resins are scalable, cost-efficient, and shorten the processing time while achieving high recovery rates [19,20]. Given the diverse physicochemical properties of macroporous resins, selecting the most suitable resin is crucial to optimize performance for specific plant materials and target components. Understanding the adsorption and desorption behavior of these components on macroporous resins helps clarify mechanisms, refine process parameters, and design effective purification systems [20].

Although many studies on polyphenols have been reported in the past two decades, there is limited information on the use of macroporous resins to specifically purify cocoa extracts for the optimal enrichment of flavonoids. Therefore, the aim of this study was to investigate different macroporous resins for the purification and enrichment of flavonoids in a cocoa extract. For in-depth characterization and optimization options, the following parameters were investigated: screening different macroporous resins, determining adsorption and desorption kinetics, varying adsorption and desorption temperatures, and optimizing elution concentrations for desorption/adsorption isotherms. The extracts were characterized in terms of dry matter content, protein content, and polyphenol content, as expressed by the total phenolic content (TPC) and the total flavonoid content (TFC). Special emphasis was given to the composition with regard to the degree of polymerization (DP) of the procyanidins.

## 2. Results

### 2.1. Screening of Macroporous Adsorption Resins for the Purification of Cocoa Flavonoids

To evaluate the purification of cocoa flavonoids from aqueous clarified cocoa extracts (cCE), the adsorption and desorption capacities of seven different macroporous resins (XAD-2, XAD-4, XAD-7, XAD-7HP, XAD-16, SP207, and HP2-MG) were characterized. The resins investigated differ in their polarity, pore structure, specific surface area, and polymer matrix, which have a significant influence on their sorption performance. XAD-2, XAD-4, and XAD-16 are non-polar polystyrene-based resins with large surface areas, while XAD-7HP, SP207, and HP2-MG are polymeric resins with medium-to-high polarity and partially acrylic-based matrices [6,21]. The properties of the resins used are listed in Section 3 Materials and Methods under Section 3.1.2 Adsorbents and Pretreatment.

**Figure 1:**
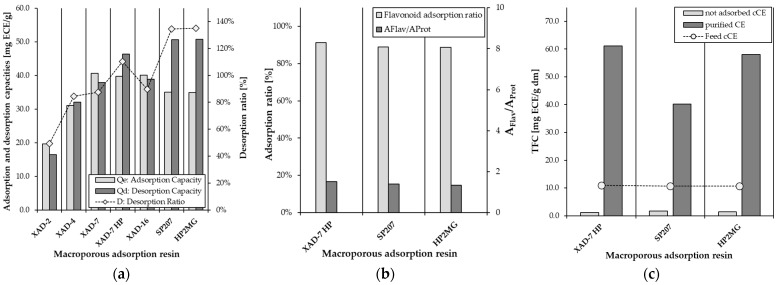
(**a**) The static adsorption and desorption capacities and desorption ratio of different macroporous resins of flavonoids at 20 ± 2 °C and eluted with 70% hydroalcoholic solution. (**b**) The static adsorption ratio and ratio of flavonoids to protein adsorption of the three best-performing macroporous resins at 20 ± 2 °C. (**c**) Total flavonoid content (TFC, mg ECE/g dm), the feed solution (cCE), and the unadsorbed cCE and purified cCE of the three best macroporous resins at 20 ± 2 °C. Adsorption and desorption capacities are expressed as the total flavonoid content (TFC, mg ECE/g resin), and the adsorption and desorption ratios are in percentages (%).

For all resins, the desorption capacities were similar or slightly higher than the adsorption capacities, except for SP207 and HP2-MG, where the desorption capacity was significantly higher. The desorption capacity and desorption ratio of the resins XAD-7HP, SP207, and HP2-MG (46.3, 50.6, and 50.8 mg ECE/g resin) were higher than those of the other resins (Figure 1a).

The adsorption and desorption capacities of macroporous resins are typically influenced by various factors, including particle size, porosity, pore radius, surface area, and the chemical structure of the polyphenols [22]. Therefore, it was assumed that the macroporous resins XAD-7HP, SP207, and HP2-MG would have had a better adsorption capacity, surface area, and pore radius than the other four resins, which had a lower adsorption capacity. These results were similar to those reported in other studies, where both the pore radius and surface area of the resins were found to be important for flavonoid adsorption [15,22,23,24,25,26]. Buran et al. [27] describe the enrichment of anthocyanins and phenolic compounds from blueberries. They achieved a desorption rate of up to 119.9% using the XAD761 resin for anthocyanins and up to 114.9% using the FPX66 resin for phenolic compounds. They explained that resins with larger particle sizes tend to have higher mass transfer rates and can transfer more material to and from the resin, increasing the desorption rate and recovery [27]. Although a positive correlation between a larger particle size and an increased mass transfer rate is described in the literature [15,22,23,24,25,26], the superior sorption performance of the resins mentioned in the present study cannot be explained by their particle size. XAD-7HP, SP207, and HP2-MG are not among the tested resins with the largest particle size, see Section 3.1.2.

Phenolic compounds are mainly adsorbed onto macroporous resins through hydrogen bonding, van der Waals forces, and π–π interactions, influenced by both the chemical structure of the phenolics and the resin’s physical properties [21,24]. These interactions enhance the selectivity of the adsorption process [28]. Consequently, the overall adsorption performance is determined by the synergistic effects of both the chemical composition and the physical properties of the resins [29]. The high desorption performance, especially for XAD-7HP, SP207, and HP2-MG, with up to 110.3%, 134.4%, and 135.1%, respectively, can be explained by the reversibility of these bonds. The high desorption performance, especially for XAD-7HP, SP207, and HP2-MG, with up to 110.3%, 134.4%, and 135.1%, respectively, can be explained by the reversibility of these bonds. The use of 70% hydroalcoholic ethanol destabilizes the hydrogen bonds between the resin and the flavonoids due to its lipophilic and polar properties. Ethanol weakens hydrophobic interactions, while the water content facilitates the dissolution of polar substances, thereby aiding desorption. Overall, this promotes the efficient desorption of flavanols from the resin’s surface [30]. Therefore, the combination of a suitable pore structure, a large polar surface, and reversible physical interactions with the flavonoids seems to contribute significantly to the efficiency of adsorption and desorption.

In addition to the adsorption and desorption capacities, the ratio of flavonoids to protein adsorption (A_Flav_/A_Prot_) is an important criterion for defining the optimum resin. Figure 1b shows the adsorption ratios of the three resins XAD-7HP, SP207, and HP2-MG with the highest adsorption capacities and consequent performance. It can be seen that their ratios were very similar to each other, with values of 1.52, 1.39, and 1.34, respectively. The XAD-7HP resin showed the best compromise between polyphenol adsorption and protein removal in the non-adsorbed clarified cocoa extract (n.a. cCE).

Another criterion used to select the optimum resin from the materials analyzed was the flavonoid content in the purified cocoa extract (pCE) in relation to the dry matter (dm). The XAD-7HP resin had the highest flavonoid content with 61.1 mg ECE/g dm, followed by HP2-MG and SP207 with 58.1 mg ECE/g dm and 52.8 mg ECE/g dm, respectively.

**Figure 2:**
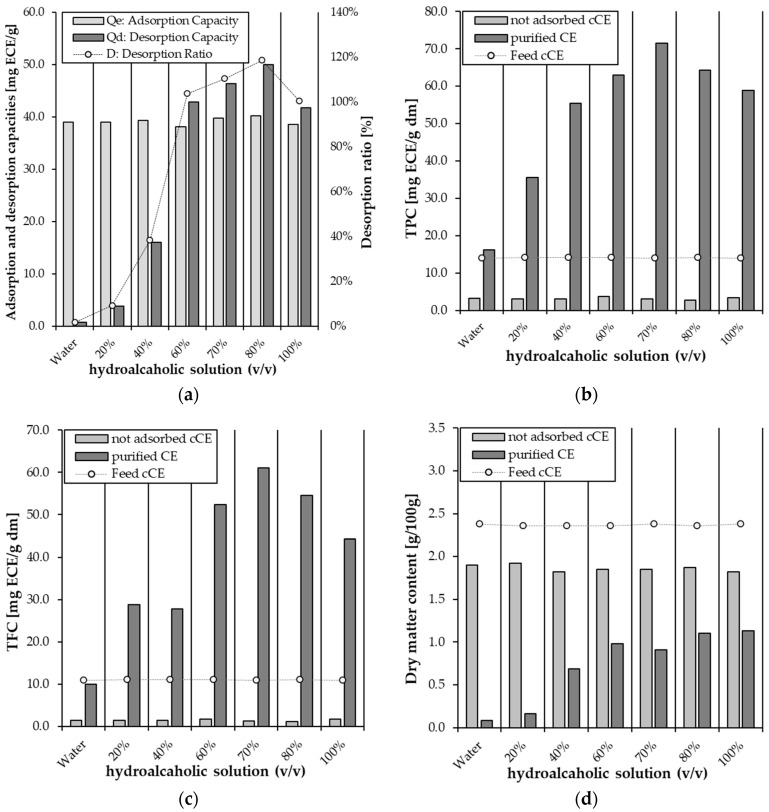
(**a**) The static adsorption and desorption capacities and the desorption ratio of the elution of flavonoids on the XAD-7HP resin at 20 ± 2 °C and with different concentrations of hydroalcoholic solutions. Adsorption and desorption capacities are expressed as the total flavonoid content (TFC, mg ECE/g resin), and the desorption ratio is expressed as a percentage (%). (**b**) Total phenolic content (TPC, mg ECE/g dm), (**c**) total flavonoid content (TFC, mg ECE/g dm), and (**d**) dry matter content (g/100 g) of the feed solution (cCE), unadsorbed cCE, and purified cCE.

XAD-7HP was selected for further experiments as it showed the highest adsorption and desorption capacities for the flavonoids in the pCE. The cCE adsorbed onto the XAD-7HP resin was eluted with different concentrations of hydroalcoholic solution (0, 20, 40, 60, 70, 80, and 100% *v*/*v*). Figure 2a shows that the desorption ratio increased rapidly with increasing ethanol concentrations in the range of 0–60%, followed by a slow increase up to 80%, then a gradual decrease. The desorption ratio reached its maximum value of 119% when the ethanol concentration reached 80%. The desorption ratio is a function of the competition between the molecular force of the polyphenols on the resin and their solubility in the eluent [11]. It can therefore be assumed that the polarity of the 80% hydroalcoholic solution is similar to the polarity of the polyphenols in the cCE. Therefore, it enhances the dissolution and elution of the polyphenols from the resin.

In addition to the adsorption and desorption capacities, when the accumulation of phenol compounds in the pCE is considered, it is noticeable that the content in the pCE was the highest with the elution of the 70% alcoholic solution. This applied to both the TPC (71.5 mg ECE/g dm) and TFC (61.1 mg ECE/g dm) content (Figure 2b,c).

It can also be observed that increasing the ethanol content also increases the dry matter content in the pCE, so it can be concluded that increasing the ethanol content also elutes other substances in addition to the polyphenols, thereby decreasing the TPC (Figure 2d). Monsanto et al. [31] and Lin et al. [24] reported similar results, also describing that a 70% hydroalcoholic solution was the most effective eluent [24,31]. Based on these results, further experiments were carried out with a 70% hydroalcoholic solution, as a high TPC in the final product is of high importance and a lower amount of ethanol is more economical.

### 2.2. Static Adsorption and Desorption Kinetics on the XAD-7HP Resin

**Figure 3:**
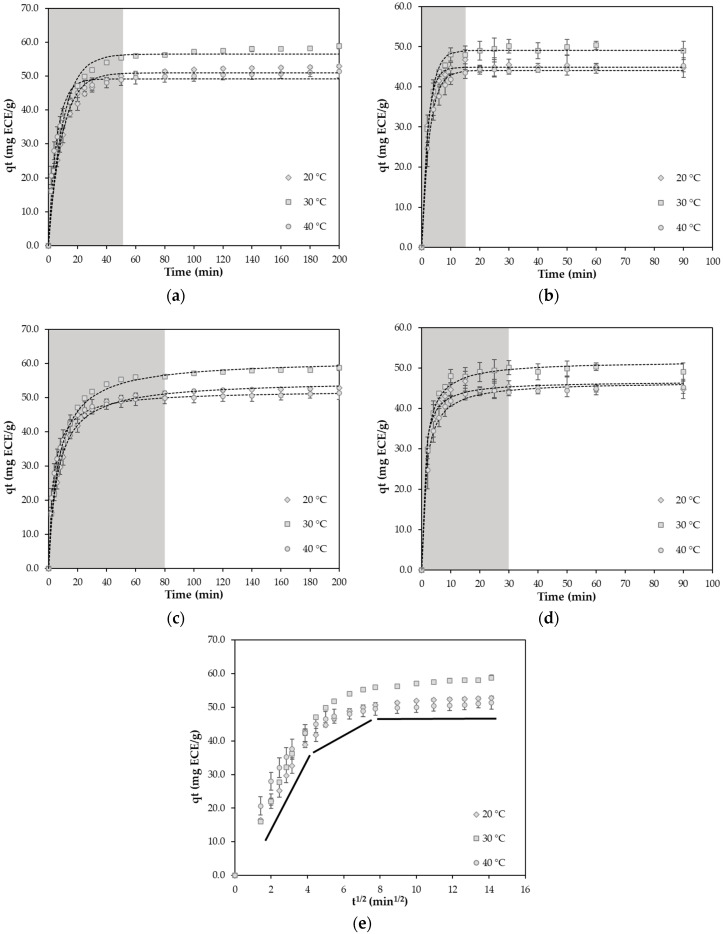
(**a**) Adsorption and (**b**) desorption kinetics of flavonoids from the cCE on the XAD-7HP resin using pseudo-first-order models; (**c**) adsorption and (**d**) desorption kinetics of flavonoids on the XAD-7HP resin using pseudo-second-order models; and (**e**) diffusion curves for adsorption. The shading in the illustrations indicates the range until equilibrium is reached. The black lines in (**e**) stand for the different adsorption stages. The $q_{t}$ was expressed as mg ECE/g. The symbols correspond to experimental data, and the dotted lines represent the calculated curves according to the selected models.

The adsorption and desorption kinetics of the XAD-7HP resin regarding the TFC in the cCE were investigated in order to determine the equilibrium contact time and optimal temperature. The experiments were carried out at temperatures of 20, 30, and 40 °C and used to generate kinetic adsorption and desorption curves. The total flavonoids adsorbed were measured as the TFC and expressed as $q_{t}$ in mg epicatechin equivalent (ECE)/g of resin at the current time point. Figure 3a,c show the kinetic curves of adsorption, and Figure 3b,d detail the desorption of flavonoids over time at the investigated temperature.

The adsorption capacity $q_{t}$ (mg ECE/g resin) increased with increasing contact time t, reaching equilibrium within 80 min. The adsorption yields ranged from 51–58% at 20 °C (52.8), 30 °C (58.8), and 40 °C (51.4). The temperature at 30 °C showed the highest $q_{t}$ values in comparison to 20 and 40 °C. Similar results were observed for desorption. Again, the desorption yield was highest at 30 °C and ranged from 44–49% at 20 °C (44.8), 30 °C (49.0), and 40 °C (45.3).

The thermodynamic aspects of flavonoid adsorption on macroporous resins, as investigated by Rodrigues et al. [32], offer a plausible explanation for the temperature-dependent behavior of the $q_{t}$ values, as it is an exothermic process. At 30 °C, the increased molecular motion enhances the diffusion of flavonoids into the resin pores, resulting in higher adsorption. In contrast, at 40 °C, the thermal destabilization of the binding interactions becomes predominant, leading to a decrease in adsorption efficiency [32,33,34]. This thermally induced behavior also influences desorption efficiency. Under conditions such as elevated temperatures or changes in solvent polarity, flavonoids may become ionized or more mobile, decreasing their affinity for the resin surface and promoting desorption [6,35]. Lin et al. [24] discovered that the enthalpy change of phenolic adsorption on XAD-7 HP was negative, indicating that the adsorption processes were exothermic. Therefore, increasing the temperature is unfavorable for the adsorption process [24]. This is emphasized by the fact that the adsorption capacity decreases with increasing temperature due to the weakening of hydrogen bonds and van der Waals forces [32,36].Therefore, room temperature was chosen for the following experiments [6,24,32,33,34,35,36,37].

The diffusion curves are shown in Figure 3e. It can be observed that the plots ($q_{t}$ versus $t^{1/2}$) are non-linear over the entire time range, indicating the manifold nature of flavonol adsorption. Thus, the adsorption process can be divided into three stages. The first stage (0–10 min) is boundary layer diffusion. The second stage (10–60 min) describes the gradual stage, where intraparticle diffusion is rate-limited. The third stage (60–200 min) belongs to the final equilibrium stage.

The experimental data in Figure 3 were fitted to the pseudo-first-order and pseudo-second-order models. The models were analyzed to explain the adsorption behavior and mechanism of the selected resin. Table 1 summarizes the parameters derived, such as the correlation coefficient and dynamic parameters. For all temperatures tested, the R^2^ values (0.977–0.934) observed for the pseudo-first-order models were lower than those (0.995–0.992) for the pseudo-second-order models. According to these values, the sorption kinetics followed a pseudo-second-order model, which has also been reported by other authors who also investigated the adsorption of total flavonoids present in various plants on different resins [15,16]. This indicates that two or more rate-limiting steps, such as external diffusion, interfacial diffusion, and intraparticle diffusion, may have controlled the adsorption process [38,39].

### 2.3. Static Adsorption Isotherms on the XAD-7HP Resin

Furthermore, the Langmuir and Freundlich isotherms were used to interpret the equilibrium relationship between the solutes and the adsorbents in the present study. The adsorption isotherm is the distribution of the adsorbed molecules between the liquid phase and the solid phase when the adsorption process reaches an equilibrium state [40]. While the Langmuir isotherm is the best-known and most commonly used model to describe the adsorption behavior of the monomolecular layer, the Freundlich model is widely used to describe the adsorption behavior of both monomolecular and multimolecular layers [27,38].

**Figure 4:**
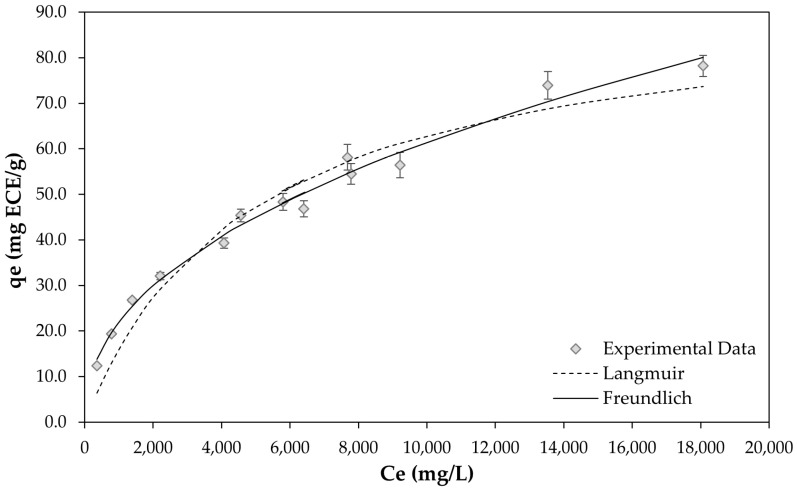
Adsorption isotherm of flavanols (expressed as the sum of DP1–DP7) from the cCE on the XAD-7HP resin with the Langmuir and Freundlich models. The symbols correspond to experimental data, and the dotted lines represent the calculated curves according to the selected models.

Figure 4 shows the adsorption isotherms of flavanols on the XAD-7HP resin at a temperature of 20 °C. Here, the flavanols, especially the oligomeric procyanidins, were measured using ultra-performance liquid chromatography (UPLC) and expressed as the sum of the degree of polymerized (DP) with a length of 1–7 molecules.

The figure shows that both the Langmuir and Freundlich models fitted the equilibrium data well, with high correlation coefficients (Langmuir: 0.94453, Freundlich: 0.9871). However, the correlation coefficient estimated from the Freundlich equation was only slightly higher; thus, the adsorption process fitted better with the Freundlich isotherm model. This suggests that the adsorption of flavanols on XAD-7 HP follows a multimolecular layer adsorption mechanism. Furthermore, the Freundlich model assumes that increasing the adsorbate concentration leads to a corresponding increase in adsorption on the adsorbent surface, reflecting multimolecular layer adsorption on heterogeneous surfaces [32].

The parameter $R_{L}$ was calculated for the Langmuir model and 1/n for the Freundlich model. The $R_{L}$ value indicates the shape of the isotherm, which is either unfavorable ($R_{L}$ > 1), linear ($R_{L}$ = 1), favorable (0 < $R_{L}$ < 1), or irreversible ($R_{L}$ = 0) [7]. The $R_{L}$ value was less than one, indicating that the adsorption of flavanols on the selected XAD-7HP resin was a favorable process (Table 2). In order to determine the adsorption intensity or the heterogeneity of the surface, the value of 1/n was measured [41]. Adsorption is considered difficult when the value of 1/n is greater than one [42]. 

As shown in Table 2, the 1/n value was less than one, indicating the favorable adsorption of flavanols on the XAD7-HP resin. This corresponds to the results for $R_{L}$.

### 2.4. Dynamic Adsorption and Desorption of cCE Flavanols on the XAD-7HP Resin

In general, when the adsorption reaches the breaking point, the adsorption affinity decreases or even disappears, and the solutes are released from the resin. Usually, the adsorption capability is reached and flavanols are leached when the flavanol concentration in the non-adsorbed effluent is 10% of the initial flavanol content ($C/C_{0}$ = 0.10) [38].

**Figure 5:**
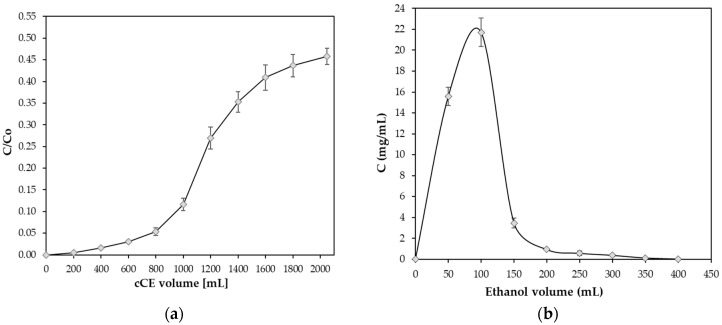
Dynamic breakthrough (**a**) and desorption (**b**) curves for the total flavanols of the cCE on XAD-7HP. (T = 20 °C ± 2 °C, $C_{0}$ = 1.71 mg/mL).

The dynamic breakthrough curve of XAD-7HP was created based on the ratio of the flavanol content (expressed as the sum of flavanols DP1–DP7) of the elution volume to the flavanol content of the initial cCE extract ($C/C_{0}$) and the elution volume. There was no obvious breakthrough of flavanols in the effluent before 800 mL cCE (Figure 5a). However, between 800 mL and 1 L, a breakthrough was observed, with $C/C_{0}=0.09$ at 800 mL and $C/C_{0}=0.20$ at 1 L. Afterwards, it was observed that the adsorption increased smoothly. In addition, at approximately 2.05 L cCE, a column overload of $C/C_{0}=0.78$ was detected. This indicates that under experimental conditions, the flavanols can be efficiently removed by the XAD-7HP resin until breakthrough occurs. The dynamic desorption curve for XAD7-HP was plotted from the elution volume for the desorption and the flavanol content of the desorbed solution. Figure 5b shows that the total flavanols were completely desorbed after an elution volume of 400 mL of 70% hydroalcoholic solution. All desorbed solutions were combined and analyzed by UPLC for their flavanol content, expressed as the sum of DP1–DP7. The cocoa flavanol content of the combined desorbed solution was 456.05 mg/mL dm. This represents a 5.03-fold increase compared to the original flavanol value of the cCE extract of 90.72 mg/mL dm (Table 3).

## 3. Materials and Methods

### 3.1. Adsorption and Desorption Experimental Set-Up

#### 3.1.1. Sample Preparation

The aqueous cocoa extract (CE) was provided by the company Oro de Cacao AG (OdC), Freienbach, Switzerland. OdC’s patented cold extraction process enables continuous, gentle processing of the cocoa beans through the process steps of wet grinding, cold extraction, and centrifuge technology with the help of a 3-phase decanter. The water phase obtained was then further purified using a centrifuge (industrial scale: capacity 2.5 m^3^/h) in order to further reduce the content of solid matter and fat in the phase [43,44]. The purified water phase (aqueous CE) contains proteins, carbohydrates, and fibers, as well as water-soluble compounds such as polyphenols and alkaloids [45]. The amount of cocoa extract used in the experiments was taken directly from the daily production of the cocoa processing plant and stored at −20 °C in 20 L buckets, with a total of 60 L.

The CE was then filtered in the laboratory using dynamic crossflow filtration with 0.2 µm ceramic membrane discs at 10 °C with a TMP of 0.8 bar. The clarified cocoa extract (cCE) was stored in batches at −20 °C until the adsorption tests.

#### 3.1.2. Adsorbents and Pretreatment

Seven non-functionalized food-grade polymeric adsorbents were used: Amberlite^®^ XAD-2, XAD-4, XAD-7, XAD-7HP, XAD-16, Sepabeads^TM^ SP207, and Diaion^®^ HP2-MG, which were purchased from Sigma-Aldrich Chemie GmbH (Buchs, Switzerland). The physical properties of these resins are summarized in Table 4. To remove monomers and porogens trapped in the pores, the resins were pretreated by soaking in 96% ethanol at a resin/solvent ratio of 1:10 for 24 h. The resins were then washed several times with deionized water, soaked in 0.1 M sodium hydroxide for a further 6 h, and then washed with deionized water until the pH was neutral. The resins were then soaked in 0.1 M hydrochloric acid for another 6 h and washed again with deionized water until the pH value was neutral. The resins were dried (70 °C) for 24 h in a universal drying oven (FD-S 115, Binder GmbH, Tuttlingen, Germany) and soaked in ethanol until use. Before use, the resins were washed with water and dried at 70 °C for 1 h.

#### 3.1.3. Dynamic Adsorption and Desorption Experiments with a Glass Column

The screening experiments of the macroporous adsorption resins were carried out in a glass column (internal diameter of 20 mm) with a length of 400 mm. The glass column was filled with 6 g of pretreated and dried adsorption resin. To regenerate the resins, it was first rinsed with 500 mL of deionized water, and then 100 mL of the cCE was added to the top of the resin in the glass column. To remove non-adsorbed components (n.a. cCE), the column was then rinsed with purified water. After the washing step, the desorption was carried out with an elution volume of 50 mL of 70% hydroalcoholic solution, collected as an eluate sample, and named pCE. Even though methanol could provide higher elution yields than ethanol [13], a food-grade solvent was preferred. All experiments were performed at room temperature (20 °C ± 2 °C). Samples were taken from the adsorption, wash, and desorption steps, and the TPC, TFC, dry matter content, and Bradford assay for protein analysis were performed directly on the samples.

The resin adsorption capacity, $Q_{e}$ (mg ECE/g), and adsorption ratio (%) were calculated using Equations (1) and (2) [46]:

(1) $$Q_{e}=\frac{\left(C_{0}-C_{e}\right)V_{i}}{W}$$

(2) $$A\;\left(\%\right)=\frac{\left(C_{0}-C_{e}\right)}{C_{0}}*100$$

$Q_{e}$ is the adsorption capacity (mg ECE/g wet resin), $C_{0}$ and $C_{e}$ are the concentrations of phenols in the aqueous cocoa extract (mg/ECE mL) before and after adsorption, respectively, $V_{i}$ is the volume of the initial sample solution (mL), and W is the weight of the wet resin (g).

The desorption capacity (mg ECE/g) and desorption ratio (%) were calculated according to Equations (3) and (4) [46]:

(3) $$Q_{d}=\frac{C_{d}V_{d}}{W}$$

(4) $$D\left(\%\right)=\frac{C_{d}V_{d}}{\left(C_{d}-C_{e}\right)V_{i}}*100$$

$Q_{d}$ is the desorption capacity (mg ECE/g wet resin), $C_{d}$ is the concentration of phenols in the desorption solution (mg ECE/mL), $V_{d}$ is the volume of the desorption solution (mL), W is the weight of the wet resin (g), D is the desorption ratio, and $C_{0}$, $C_{e}$, and $V_{i}$ are the same as described above.

As XAD-7HP proved to be the best resin with the highest adsorption and desorption capacities for flavonoids of the aqueous cCE, further tests were carried out with this resin. To investigate desorption, different concentrations of ethanol (20%, 40%, 60%, 70%, 80%, and 100% m/m) were compared in terms of their effect on flavonoid desorption. As before, samples were taken from the adsorption, wash, and desorption steps, and the TPC and TFC assays were performed directly on the samples.

#### 3.1.4. Static Adsorption and Desorption Kinetics Experiments

**Figure 6:**
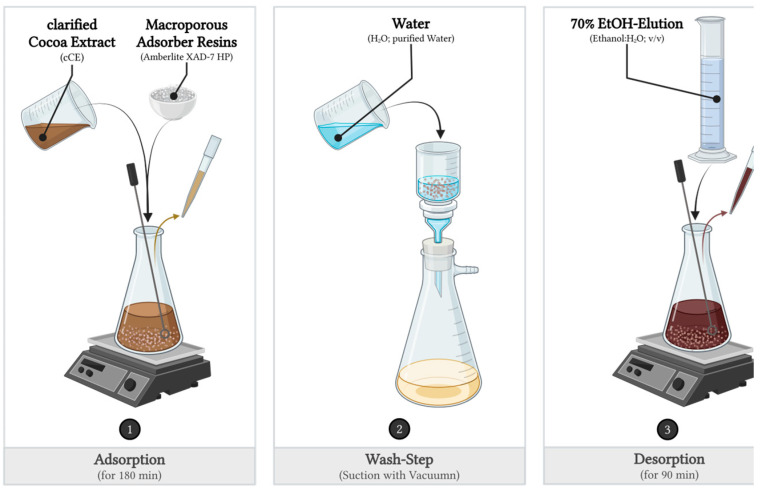
Scheme of the adsorption and desorption kinetics experiments. The experiments were carried out with 300 mL of the cCE on 12 g of adsorbent resin and 210 mL of desorption solution. The experiments were conducted at temperatures of 20, 30, and 40 °C. Created in BioRender Beeler, N. (2025). https://BioRender.com.

Adsorption kinetics experiments on the preliminary selected resins and the elution of the 70% hydroalcoholic solution were conducted in 300 mL Erlenmeyer flasks by adding 12 g of the hydrated resin to 300 mL of the cCE. The mixture was stirred at 20 °C ± 2 °C and 180 rpm for 3 h with a propeller stirrer (RZR 2020 with PR 30, Heidolph Instruments GmbH & Co. KG, Schwabach, Germany). Samples of 15 mL were taken at time points of 0, 2, 4, 6, 8, 10, 15, 20, 25, 30, 40, 50, 60, 80, 100, 120, 140, 160, and 180 min to determine the TFC. To evaluate the desorption kinetics, the resin from the adsorption experiments was washed with 300 mL of purified water and then eluted with a 70% hydroalcoholic solution. Again, 15 mL samples were taken at 0, 2, 4, 6, 8, 10, 15, 20, 25, 30, 40, 50, 60, and 90 min to determine the TFC. A schematic diagram of the experiment is shown in Figure 6.

For the calculation of the adsorption capacity at time t, Equation (5) was used:

(5) $$q_{t,\;\;ads}=\frac{\left(C_{0}-C_{a}\right)\left(V_{0}-\sum_{1}^{t}V_{sample,t-1}\right)}{{XM}_{ads}}$$

where $q_{t,\;ads}$ is the amount of adsorbed flavanols at time t, $C_{a}$ is the concentration of flavanols at time t, and $\sum_{1}^{t}V_{sample,\;t-1}$ is the total volume of the sample removed from the cCE solution at time (t−1).

The desorption capacity at time t was calculated using Equation (6):

(6) $$q_{t,\;des}=\frac{\left(C_{d}\right)\left(V_{0}-\sum_{1}^{t}V_{sample,t-1}\right)}{{XM}_{ads}}$$

where $q_{t,\;des}$ is the amount of desorbed flavanols at time t, $C_{d}$ is the concentration of flavanols at time t, and $\sum_{1}^{t}V_{sample,\;t-1}$ is the total volume of the sample removed from the desorption solution at time (t−1).

The experimental data obtained were fitted to the pseudo-first-order model (Equation (6)) or the pseudo-second-order model (Equation (7)). The total phenolics adsorbed are measured by the variable $q_{t}$ and expressed as mg ECE/g:

(7) $$\frac{d_{q}}{d_{t}}=k_{1}\left(q_{e}-q_{t}\right)$$

(8) $$\frac{d_{q}}{d_{t}}=k_{2}{\left(q_{e}-q_{t}\right)}^{2}$$

The particle diffusion kinetics model was expressed as:

(9) $$q_{t}=k_{d}*t^{\left(1/2\right)}+C$$

$q_{e}$ and $q_{t}$ are the adsorption and desorption capacities at equilibrium and at time t, respectively. $k_{1}$ $\left({min}^{-1}\right)$, $k_{2}$ $\left({g\;mg}^{-1}\;{min}^{-1}\right),$ and $k_{d}$ are the corresponding rate constants for the PFO, PSO, and particle diffusion models. C is the constant in the particle diffusion kinetics model.

#### 3.1.5. Static Adsorption Isotherms Experiments

The static adsorption isotherms were carried out in a 100 mL Erlenmeyer flask at room temperature (22 °C). For this purpose, 50 mL of the sample was placed in the Erlenmeyer flask, 2 g of pretreated resin was added, and the flask was sealed with PARAFILM^®^. The sample was stirred with a multi-position magnetic stirrer (RT 10, IKA-Werke GmbH & Co. KG, Staufen, Germany) at 150 rpm for 5 h. The permeate was concentrated with a rotary evaporator (Rotavapor^®^ R-300, BÜCHI Labortechnik AG, Flawil, Switzerland) and 13 dilutions were made with the following concentrations: 1.39, 2.55, 3.62, 5.10, 7.65, 8.37, 9.92, 10.19, 12.74, 12.55, 13.94, 19.62, and 24.52 mg/mL.

The adsorption isotherm is essential for optimizing adsorption mechanisms and describing the surface properties and capacities of adsorbents, as it explains how the adsorbate interacts with the adsorbent [47]. Thus, the adsorption isotherm is the distribution of adsorbate molecules between the liquid phase and the solid phase when the adsorption process reaches an equilibrium state [40].

While the Langmuir equation is the best-known and most commonly used model to describe the adsorption behavior of the monomolecular layer, the Freundlich model is widely used to describe the adsorption behavior of both monomolecular and multimolecular layers [38,48]. The Langmuir isotherm has been calculated using the equation given in Equation (10):

(10) $$Q_{e}=\frac{Q_{max}K_{L}C_{e}}{1+K_{L}C_{e}}$$

where $Q_{e}$ is the amount of adsorbed flavanols and $Q_{max}$ is the maximum adsorption capacity of the flavanols.

The dimensionless separation factor of the equilibrium parameter can be used to express the favorable nature of the adsorption, given by Equation (11) [49]:

(11) $$R_{L}=\frac{1}{\left(1+K_{L}C_{e}\right)}$$

$K_{L}$ is the Langmuir constant and $C_{e}$ is the adsorbate concentration of the solution.

The Freundlich isotherm was calculated using Equation (12):

(12) $$Q_{e}=K_{F}C_{e}^{1/n}$$

$K_{F}$ represents the Freundlich constant, which is an indicator of adsorption capacity, and 1/n is the heterogeneity factor, which is an empirical constant that is dependent on the temperature and the adsorption system [50].

#### 3.1.6. Dynamic Adsorption and Desorption Experiments

The dynamic adsorption and desorption properties of XAD-7HP were evaluated using a glass column (with an internal diameter of 20 mm) with a length of 400 mm and carried out at a temperature of 20 ± 2 °C. The glass column was filled with 24 g of pretreated and dried adsorption resin. To regenerate the resins, it was first rinsed with 1 L of deionized water and then 1.9 L of the cCE was added to the top of the resin in the glass column. To determine the breakthrough curve, a sample of the n.a. cCE was taken every 200 mL and analyzed to obtain the TFC. Afterwards, the column was rinsed with 400 mL of purified water to remove the remaining non-adsorbed components.

To evaluate the dynamic desorption curve for XAD7-HP, the adsorbed components were desorbed with 400 mL of 70% hydroalcoholic solution, and a sample of the eluate (pCE) was taken every 50 mL to analyze the TFC.

### 3.2. Chemicals and Reagents

The solvents and reagents used for the analyses of the experiments were purchased from Sigma-Aldrich Chemie GmbH (Buchs, Switzerland), unless otherwise stated.

For Bradford protein analysis, a Coomassie (Bradford) Protein Assay Kit and bovine serum albumin (BSA) as a standard protein were acquired from VWR International GmbH (Dietikon, Switzerland).

Folin–Ciocalteu’s phenol reagent (2 N), Na_2_CO_3_ (sodium carbonate; anhydrous, ≥99.5%), NaNO_2_ (sodium nitrite; ≥99.0%), AICI_3_ (aluminium chloride; anhydrous, ≥99.0%), NaOH (sodium hydroxide solution (1 N)), (−)-epicatechin, and absolute ethanol were purchased from Sigma-Aldrich Chemie GmbH (Buchs, Switzerland).

In addition, the following chemicals were used for flavonoid extraction and analysis (all HPLC-grade): acetone, acetonitrile, acetic acid (glacial), and methanol (Sigma-Aldrich Chemie GmbH, Buchs, Switzerland). Purified water was produced with the water purification system of Merck Milli-Q (0.22 µm) (Simplicity UV, Merck & Cie KmG, Schaffhausen, Switzerland). Cocoa Extract Calibrant (NIST Reference Material No. 8403), purchased from the National Institute of Standards and Technology (NIST, Gaithersburg, MD, USA), was used to prepare standards for identification and quantification.

### 3.3. Physicochemical Analysis of the Extract, Supernatant, and Filtrate

To verify the purification performance of the macroporous resins, the following analyses were carried out: dry matter content, protein content, TPC, TFC, and individual flavanols with DP1–7.

#### 3.3.1. Dry Matter Content Based on a Halogen Dryer Method

The dry mass was measured using a halogen dryer (Moisture Analyzer HC103, Mettler Toledo GmbH, Greifensee, Switzerland), in accordance with the method described by Beeler et al. [51].

#### 3.3.2. Protein Content Using the Bradford Method

The Bradford method was performed using the Coomassie Plus (Bradford) Assay Kit according to Bradford [52] and the adaptations of Beeler et al. [51]. The sample solution was measured against water as a blank at a wavelength of 595 nm using a UV/vis spectrophotometer (Genesys^TM^ 10S, Thermo Fisher Scientific AG, Reinach, Switzerland). The protein concentration of each sample was determined with a calibration line; a third-order polynomial regression line of y = 0.0392 × x^3^ − 0.0009 × x^2^ + 0.0349 × x − 0.0005 (R^2^ = 0.99986) was obtained. BSA was used as a calibration standard, and the results were expressed as milligrams of BSA equivalent per milliliter (mg BSA/mL).

#### 3.3.3. Total Phenolic Content (TPC) Using the Folin–Ciocalteu Assay

The photometric method for determining the TPC is used in the food industry quite often. Therefore, this assay was used to assess the filtration performance. However, this method also detects other reducing substances, such as sugars and small portions of proteins, in addition to polyphenols, as these substances also react with the Folin–Ciocalteu reagent. The assay was carried out according to Blois [53] and the adaptations of Pedan et al. [54].

Due to high reactivity, the cocoa extract and the retentate were diluted 1:100, and the filtered cocoa extract (permeate) was diluted 2:100 with deionized water. One aliquot (1 mL) of the diluted sample was mixed with 1 mL of the Folin reagent (2 N reagent diluted 1:3 with deionized water), and after the addition of 2 mL of deionized water, it was incubated for 3 min at room temperature. Afterwards, 2 mL of anhydrous sodium carbonate solution (20% Na_2_CO_3_, *w*/*v*) was added. The solution was kept for 2 h at room temperature for color formation, and the absorbance of the blue-colored samples was measured at a wavelength of 750 nm with a UV/vis spectrophotometer (Genesys^TM^ 10S, Thermo Fischer Scientific AG, Reinach, Switzerland) against a blank containing the same reagents and 1 mL of deon. water. Epicatechin (EC) was used as a calibration standard, and the results were expressed in milligrams per (−)-epicatechin equivalent per milliliter (mg ECE/mL). A linear regression line of y = (x − 0.0681)/16.707 (R^2^ = 0.9977) was obtained.

#### 3.3.4. Total Flavonoid Content (TFC) Using the Aluminum Chloride Assay

Besides the determination of the TPC, the TFC gives an indication of the phenolic compound composition. The photometric method was carried out according to the described method of Zzaman et al. [55], with the adjustments of Pedan et al. [54].

The cocoa extract samples were diluted in ratios of 4:100 and 8:100, respectively. Further, 1 mL of the diluted sample was mixed with 4 mL of deionized water and 0.3 mL of sodium nitrite solution (5% NaNO_2_, *w*/*v*) and incubated for 6 min at room temperature. Subsequently, 0.3 mL of aluminum chloride (10% AICI_3_, *w*/*v*) was added to the solution and incubated for another 6 min. After the addition of 2 mL of 1 M NaOH and 2.4 mL of deionized water, the pink-colored solution was incubated for another 15 min, and the absorbance was measured at a wavelength of 510 nm. Epicatechin (EC) was used as a calibration standard, and the results were expressed in milligrams per (−)-epicatechin equivalent per milliliter (mg ECE/mL). A linear regression line of y = (x − 0.0115)/3.4461 (R^2^ = 0.9997) was obtained.

#### 3.3.5. Content of Individual Flavanols Using UPLC Analysis

The flavonoid content was determined using the official AOAC 20 May 2020 for the determination of flavanol and procyanidin oligomers (DP 1–7) in cocoa-based products. The flavanols and procyanidins were extracted using an acidified aqueous acetone solvent system (AWAA; acetone/water/acetic acid, 70:30:1, *v*/*v*/*v*). The obtained extracts were then filtered through a 0.22 µm syringe filter (VWR^®^ Syringe Filter, hydrophobic PTFE, 13 mm, 0.22 µm, VWR International GmbH, Dietikon, Switzerland) and transferred to chromatography vials for HPLC analysis.

*Extraction*—To extract the flavanols and procyanidins, 0.5 mL of the cocoa extract sample was transferred to a 5 mL disposable centrifuge tube and mixed with 4.5 mL of acidified aqueous acetone (AWAA). After a brief period of manual shaking, the mixture was placed in a sonication bath at 50 °C for five minutes, followed by centrifugation at 3068× *g* for five minutes. Approximately 1 mL of the supernatant (diluted extract or permeate) was then filtered through a 0.2 µm syringe filter into an HPLC vial.

*Chemical analysis*—The compound composition was analyzed using ultra-performance liquid chromatography with fluorescence detection (UPLC-FLR) on a Waters Acquity UPLC system. This system was equipped with a sample manager (FTN-H), a quaternary solvent manager (QSM), and an Acquity UPLC-FLR detector (Waters AG, Baden-Dättwil, Switzerland). The injection volume was 2 µL.

*Chromatographic conditions*—Separation was performed on a torus diol column (100 × 3.0 mm i.d., 1.7 µm, 130 Å; Waters AG, Baden-Dättwil, Switzerland) at a flow rate of 1 mL/min and a column temperature of 50 °C. The autosampler was maintained at 5 °C. Prior to analysis, the column was equilibrated with a 50:50 mixture of solvents A and B for at least 10 min.

*Solvents and gradient*—Mobile phase A consisted of acetonitrile and acetic acid (98:2, *v*/*v*), while mobile phase B consisted of methanol, water, and acetic acid (95:3:2, *v*/*v*/*v*). The gradient program was as follows: 0–0.37 min: 0% B; 0.37–10.03 min: linear increase to 45% B; 10.03–10.28 min: increase to 95% B; 10.28–12.63 min: 95% B; 12.63–12.73 min: return to 0% B. The total run time was 13.10 min, with a 3 min equilibration period.

*Fluorescence detection*—The compounds were detected using fluorescence at an excitation wavelength of 230 nm and an emission wavelength of 321 nm.

*Quantification*—Quantification was performed using NIST’s cocoa extract standard (NIST Reference Material No. 8403) to determine retention times and generate calibration curves. Linear regression was then used to quantify each degree of polymerization (DP) group. Information on the calibration and detection parameters as well as the chromatogram of the NIST cocoa flavanol standard can be found in the supplementary material. Data acquisition and analysis were performed using Empower 3 software (Waters AG, Baden-Dättwil, Switzerland). Results are expressed as milligrams per gram of dry matter (mg/g dm).

## 4. Conclusions

In the present study, different macroporous adsorption resins were screened with regard to their adsorption and desorption capacities for cocoa flavanols from an aqueous clarified cocoa extract. The results demonstrate that, among the tested resins, the macroporous resin Amberlite^®^ XAD-7HP was best-suited to purifying the cocoa flavanols. Regarding the static adsorption and desorption kinetics of the total flavonoids for the cCE on XAD-7HP, the pseudo-second-order model best fitted the equilibrium data. Furthermore, the Langmuir and Freundlich isotherms were used to interpret the equilibrium relationship between the solutes and the adsorbents in the present study. The Freundlich isotherm model described the data slightly better than the Langmuir model, suggesting the formation of a multimolecular layer.

This study underlines the promising potential of cocoa flavanol enrichment using macroporous adsorber resins. Furthermore, the results of this study indicated that good yields can be achieved with the XAD-7HP resin with a previous clarification process with dynamic crossflow filtration. However, further investigations are needed to investigate the adsorption and desorption process for future process upscaling. With regard to process engineering, column height and diameter would be very important parameters. These findings may be transferable to other complex flavanol-rich extracts and represent a promising approach for the development of flavanol enrichment processes, whereby the isolated flavanols can contribute to the attractive valorization of a by-product.

## Figures and Tables

**Table 1:** Regression coefficients of pseudo-first-order and pseudo-second-order models for the adsorption of total flavonoids (as mg epicatechin equivalents/g resin) from cocoa extracts.

Process	Temp. (°C)	*q_e_*_(*exp*)_ (mg/g)	Pseudo-First-Order Model			Pseudo-Second-Order Model		
			*k*_1_ (min^−1^)	*q_e_*_(*cal*)_ (mg/g)	R^2^	*k*_2_ (g/mg × min)	*q_e_*_(*cal*)_ (mg/g)	R^2^
Absorption	20	52.84	0.108	50.97	0.959	0.003	55.13	0.992
	30	58.81	0.104	56.52	0.977	0.002	61.32	0.994
	40	51.37	0.176	49.09	0.934	0.006	52.09	0.995
Desorption	20	44.81	0.504	44.85	0.964	0.023	46.77	0.940
	30	49.04	0.411	49.03	0.661	0.015	51.71	0.971
	40	45.25	0.370	44.07	0.974	0.014	46.72	0.977

**Table 2:** Isotherm parameters for flavonoid adsorption on the XAD-7HP resin. The $R_{L}$ parameter shows the average $R_{L}$ value of all concentrations.

Langmuir				Freundlich		
*q_m_* (mg/g)	*K_L_* (L/g)	*R_L_*	R^2^	1/*n*	*K_f_*	R^2^
93.61	2.05 × 10^−4^	0.524	0.944	0.448	0.996	0.987

**Table 3:** UPLC cocoa flavanol content expressed as the sum of the DP1–DP7 of the samples from the adsorption and desorption processes.

Sample	Amount [g]	Cocoa Flavanols (Sum of DP1–DP7)	Cocoa Flavanols (Sum of DP1–DP7)	Recovery Yield Flavanols	Yield Sample as Powder
		[mg/mL]	[mg/mL dm]	[%]	[%]
cCE	1900	1.71	90.72	100	100
n.a. cCE	1900	0.39	23.71	23	86
Wash Water	400	0.74	135.80	9	6
pCE	400	5.47	456.05	67	13

**Table 4:** Physiochemical properties of screened macroporous adsorption resins used for the recovery of phenolic compounds from the cCE. PS-DVB: polystyrene–divinylbenzene; B-PS: brominated polystyrene–divinylbenzene.

Resins	XAD-2	XAD-4	XAD-7	XAD-7HP	XAD-16	SP207	HP2-MG
Structure	PS-DVB	PS-DVB	Aliphatic ester	Aliphatic ester	PS-DVB	B-PS-DVB	Polymethacrylate
Polarity	Non-polar	Non-polar	Strongly polar	Strongly polar	Non-polar	Non-polar	Moderately polar
Surface area [m^2^/g]	330	725	450	500	900	630	470
Porosity [mL/g]	0.65	0.98	1.14	1.08	1.82	1.1	1.2
Pore radius [Å]	90	50	90	550	100	120	170
Particle size [mm]	0.25–0.84	0.25–0.84	0.25–0.84	0.43–0.69	0.25–0.84	0.25–0.60	0.25–0.60

## Data Availability

The data presented in this study are available on request from the corresponding author.

## Supplementary Materials

The following supporting information can be downloaded at: https://www.mdpi.com/article/10.3390/molecules30112336/s1, Table S1: Information on the calibration and detection parameters that are used for the analysis of the different compounds that are quantified, Figure S1: Chromatogram of the NIST cocoa flavanol standard to illustrate the DP groups. Further data presented in this study are available upon request from the corresponding author.
