# Supplementary material for: Purification of Flavonoids from an Aqueous Cocoa (Theobroma cocoa L.) Extract Using Macroporous Adsorption Resins
Source: Molecules. 2025 May 27;30(11):2336. doi: 10.3390/molecules30112336 (PMC12155860; doi:10.3390/molecules30112336)
Supplement: Supplementary file 1 [file molecules-30-02336-s001.zip › molecules-3620839-supplementary.pdf]

Supplementary Materials

Table S1. Information on the calibration and detection parameters that are used for the analysis of the different compounds that are quantified.

Group	retention time start	retention time stop	measurement-/ detection system	detection parameters	calibration points	calibration range [µg/mL]	calibration line	(r^2)
DP1	0.845	2.073	UPLC-FLD	Ex = 230 nm<br>Em = 321 nm	5	4.872 – 24.359	y=1.03e+05x-1.16e+05	0.999009
DP2	2.352	3.290	UPLC-FLD	Ex = 230 nm<br>Em = 321 nm	5	3.502 – 17.511	y=5.75e+04x-1.15e+05	0.994884
DP3	4.410	5.060	UPLC-FLD	Ex = 230 nm<br>Em = 321 nm	5	3.706 – 18.529	y=1.58e+04x-4.75e+04	0.980872
DP4	5.589	6.360	UPLC-FLD	Ex = 230 nm<br>Em = 321 nm	5	3.138 – 15.688	y=1.78e+04x-3.43e+04	0.992500
DP5	6.360	7.153	UPLC-FLD	Ex = 230 nm<br>Em = 321 nm	5	2.658 – 13.292	y=1.27e+04x-1.63e+04	0.996326
DP6	7.160	7.935	UPLC-FLD	Ex = 230 nm<br>Em = 321 nm	5	2.052 – 10.261	y=1.36e+04x-4.91e+03	0.994945
DP7	7.935	8.403	UPLC-FLD	Ex = 230 nm<br>Em = 321 nm	5	1.556 – 7.780	y=91.56e+04x-3.41e+03	0.948322

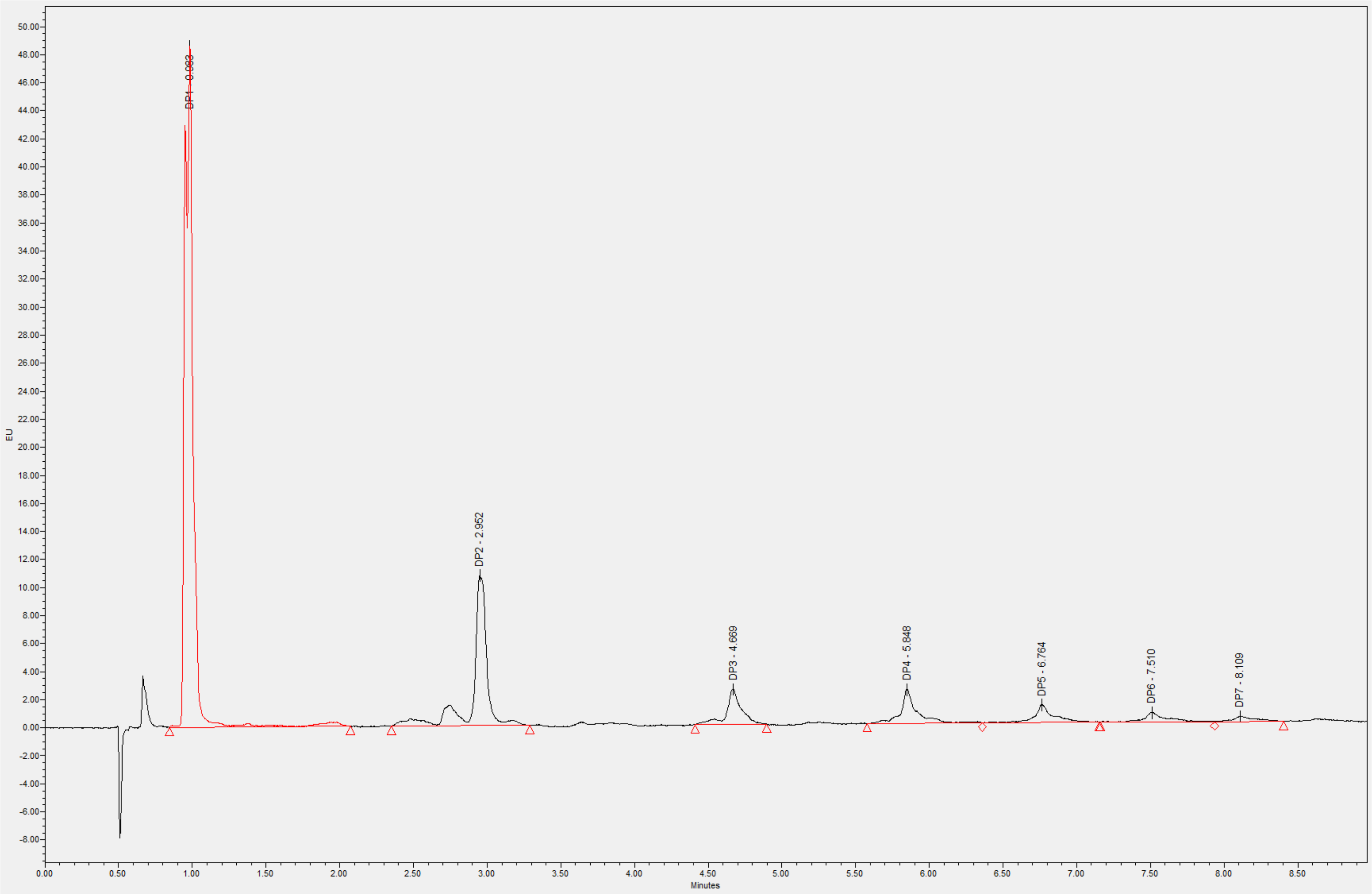

Figure S1. Chromatogram of the NIST cocoa flavanol standard to illustrate the DP groups.
